# Generation and Regeneration of Thymic Epithelial Cells

**DOI:** 10.3389/fimmu.2020.00858

**Published:** 2020-05-07

**Authors:** Abdullah S. Alawam, Graham Anderson, Beth Lucas

**Affiliations:** Institute for Immunology and Immunotherapy, University of Birmingham, Birmingham, United Kingdom

**Keywords:** thymus, thymic epithelial cell, thymic atrophy, regeneration, bone marrow transplant, immune reconstitution

## Abstract

The thymus is unique in its ability to support the maturation of phenotypically and functionally distinct T cell sub-lineages. Through its combined production of MHC-restricted conventional CD4^+^ and CD8^+^, and Foxp3^+^ regulatory T cells, as well as non-conventional CD1d-restricted iNKT cells and invariant γδT cells, the thymus represents an important orchestrator of immune system development and control. It is now clear that thymus function is largely determined by the availability of stromal microenvironments. These specialized areas emerge during thymus organogenesis and are maintained throughout life. They are formed from both epithelial and mesenchymal components, and collectively they support a stepwise program of thymocyte development. Of these stromal cells, cortical, and medullary thymic epithelial cells represent functional components of thymic microenvironments in both the cortex and medulla. Importantly, a key feature of thymus function is that levels of T cell production are not constant throughout life. Here, multiple physiological factors including aging, stress and pregnancy can have either short- or long-term detrimental impact on rates of thymus function. Here, we summarize our current understanding of the development and function of thymic epithelial cells, and relate this to strategies to protect and/or restore thymic epithelial cell function for therapeutic benefit.

## Introduction

While the bone marrow represents a major site of hemopoiesis, including hemopoietic stem cell development and maintenance as well as B-cell development, the generation of αβT cells relies upon the exit of lymphoid progenitors from the bone marrow and their entry into the thymus. Here, blood-borne thymus colonizing cells undergo a complex differentiation program that includes lineage restriction, proliferation, and T cell receptor gene rearrangement and selection. This results in the generation of a pool of self-tolerant αβTCR-expressing CD4^+^ and CD8^+^ mature thymocytes that then leave the thymus and form the peripheral T cell pool (1–3). Critically, intrathymic T cell development is a non-cell autonomous process and requires continual input from highly heterogeneous stromal cell populations that collectively form intrathymic microenvironments. Of particular importance is that such microenvironments contain both cortical and medullary regions, each consisting of, and defined by, specialized epithelial cells with differing roles (4, 5). Cortical thymic epithelial cells (cTEC) are responsible for driving immature CD4^−^CD8^−^ lymphoid progenitors toward the T cell lineage, and the subsequent positive selection of CD4^+^CD8^+^ thymocytes. In addition, and following developmental stages in the cortex, thymocytes migrate into medullary thymic areas, with medullary thymic epithelial cells (mTEC) attracting positively selected thymocytes and providing environmental cues which aid in self-tolerance mechanisms that include deletion of autoreactive thymocytes via negative selection and sublineage divergence for the generation of Foxp3^+^CD4^+^ T-Regulatory (T-Reg) cells (6, 7). Based on this, current models of intrathymic αβT cell development center around a step-wise process in which sequential interactions with cTEC and then mTEC generate and shape the αβT cell pool. Importantly, in addition to generating conventional and Foxp3^+^ T-Reg that express diverse TCR repertoires, the thymus also supports the development of innate-like T cell subsets that can be defined by their expression of restricted TCR repertoires. In the embryonic period, examples of this are the serial waves of invariant γδ-cells that seed specific peripheral tissues, while the post-natal generation of CD1d-restricted invariant NKT cells demonstrates how the thymus supports the development of multiple T cell types throughout life (8).

While TEC populations are known to be key regulators of these distinct T cell development programs, recent studies have uncovered significant new TEC heterogeneity that must be considered in relation to our understanding of microenvironmental control of T cell development (9–11). Significantly, therapeutic interventions can also be detrimental to thymus function (12, 13). Such clinical treatments include ablative preconditioning used in the treatment of cancer, which then impairs T cell mediated immune reconstitution following bone marrow transplantation. Consequently, studying thymic epithelial cells in both health and disease states is important to understand how thymus function is controlled, and how it might be manipulated for therapeutic benefit. Recently, important advances have been made in understanding the biology of thymic epithelium, including the developmental pathways that give rise to distinct cortical and medullary epithelial lineages. Furthermore, there is progress in how newly identified heterogeneity in thymic epithelium may map to functional specialization in thymic microenvironments.

## Lineage Specific Thymic Epithelial Cells

### cTEC and cTEC Heterogeneity

The thymus cortex, and in particular the cTEC that reside there, play multiple key roles in T-cell development. These events include pre-TCR mediated transition of CD4^−^CD8^−^ precursors to the CD4^+^CD8^+^ stage, and the positive selection of CD4^+^CD8^+^ cells expressing αβTCRs capable of MHC recognition. Interestingly, interactions between CXCR4 and its ligand CXCL12, a chemokine expressed by cTEC, have been reported to play a role in the regulation of both these events (Table 1). For example, CXCR4–CXCL12 interactions regulate the intrathymic positioning of T cell progenitors (14) while the maturation of pre-TCR expressing CD4^−^CD8^−^ progenitors requires CXCR4–CXCL12 to act in concert with Notch signaling in order to drive β-selection (15, 16). For later stages of thymocyte development, CXCL12 has recently been reported to act as a cortex retention factor for CD4^+^CD8^+^ thymocytes, which may enable cells to stay within the thymic cortex in order to undergo correct maturational events, including positive selection (17). Interestingly, of relevance to these studies that indicate the importance of CXCL12–CXCR4, is analysis of the expression and distribution pattern of CXCL12. Thus, analysis of CXCL12^dsRed^ knockin mice showed that CXCL12 is expressed by Ly51^+^ cTEC, and is distributed throughout the thymic cortex microenvironment (18). As such, it is not currently clear how such a broad expression pattern of CXCL12 might relate to the possibility that particular regions of the thymus cortex are specialized to support specific maturational events. Moreover, it is also important to note that deletion of CXCR4 expression using CD4^cre^, which selectively targets CD4^+^CD8^+^ thymocytes and their downstream products, did not alter thymocyte development, nor the intrathymic positioning of CD4^+^CD8^+^, CD4^+^, and CD8^+^ thymocytes (18). Thus, at stages downstream of pre-TCR mediated events, it is currently still unclear to what extent CXCR4 plays an important role. Perhaps relevant to this, CCR9 is an additional chemokine receptor expressed by CD4^+^CD8^+^ thymocytes which, via interplay with plexinD1-semaphorin3E interactions, has been reported to play a role in the intrathymic positioning of thymocytes (19). Whether such findings collectively indicate potential functional redundancy and/or hierarchy in chemokine receptors and ligands that regulate the cortex residency of CD4^+^CD8^+^ requires further investigation.

**Table 1:** Differential gene expression in cTEC and mTEC subsets.

**Molecule**	**Gene Name**	**Expression pattern**	**Reported functions in thymus**	**References**
CD205	*Ly75*	Broad within cTEC	Apoptotic cell clearance	(105)
β5t	*Psmb11*	Broad within cTEC, also TEC progenitors	Thymoproteosome component, CD8 positive selection	(31, 71, 106)
PRSS16	*Prss16*	cTEC	Thymus specific serine protease, CD4 positive selection	(107)
Delta like 4	*Dll4*	cTEC	Notch ligand, regulator of T-cell commitment and β-selection	(108)
CXCL12	*Cxcl12*	Broad within cTEC	Chemokine ligand for CXCR4, regulation of β-selection	(14–18)
CCL25	*Ccl25*	cTEC and mTEC	Chemokine ligand for CCR9, recruitment and positioning of T-cell progenitors, regulator of CD4^+^CD8^+^ thymocyte migration	(95, 109, 110)
CCRL1	*Ackr4*	cTEC and mTEC	Atypical chemokine receptor, scavenging receptor for CCL19, CCL21, CCL25	(111, 112)
LTβR	*Ltbr*	cTEC and mTEC	Ligand for lymphotoxin and light, regulator of mTEC development and thymic endothelium development. No known role in cTEC	(34, 45, 96, 97, 113, 114)
Aire	*Aire*	mTEC^hi^	Tissue restricted antigen expression, tolerance	(115, 116)
Fezf2	*Fezf2*	mTEC^hi^ and mTEC^lo^	Tissue restricted antigen expression, tolerance	(44, 45)
RANK	*Tnfrsf11a*	mTEC^hi^ and mTEC^lo^, mTEC progenitors	mTEC development	(32, 33, 38, 40, 41, 117)
OPG	*Tnfrsf11b*	mTEC^hi^	Negative regulator of mTEC	(33, 39, 41)
IL25	*Il25*	Thymic tuft cells	Regulation of intrathymic ILC and iNKT-cells	(9, 11)
IL15	*Il15*	mTEC^lo^	IL15 transpresentation, regulation of iNKT-cells	(59, 118)
IL15Rα	*Il15ra*	mTEC^lo^	IL15 transpresentation, regulation of iNKT-cells	(59)
IL7	Il7	cTEC and mTEC in adult thymus, TEC progenitors in embryonic thymus	T-cell progenitor proliferation	(70)
CCL21	*Ccl21a*	mTEC	Chemokine ligand for CCR7, regulator of cortex to medulla migration of SP thymocytes	(37, 42)
Relb	*Relb*	mTEC	mTEC progenitor development	(32, 119, 120)
SCF	*Kitlg*	cTEC	Maintenance of T cell progenitors	(121, 122)

*LTβ R, Lymphotoxin beta Receptor; Aire, Autoimmune Regulator; RANK, Receptor Activator of Nuclear Factor κ B; OPG, Osteoprotegerin; ILC, Innate Lymphoid Cell; iNKT, invariant Natural Killer T Cell; SP, Single Positive; SCF, Stem Cell Factor*.

Regarding the ability of cTEC to support MHC class I mediated positive selection of CD8^+^ T cells, processing and presentation of peptides associated with MHC-I molecules requires proteasomal degradation. The thymoproteasome is a unique type of proteasome expressed specifically by cTECs, the catalytic subunit of which is β5t (Table 1). Mice deficient in β5t have reduced positive selection of CD8^+^ thymocytes (20). cTEC restricted expression of β5t is also seen within human TECs, interestingly however analysis of humans carrying mutations within PSMB11 has not revealed any adverse effects (21). Nevertheless, β5t is a defining feature of cTEC that directly underpins at least part of their functional specialization for positive selection. Importantly, while β5t expression by cTEC in the adult thymus is an important defining feature of cTEC functionality, it is also expressed by immature TEC progenitors. This is perhaps most notable in analysis of the embryonic thymus, where fate mapping of β5t expressing cells showed that mTEC, including the Aire^+^ subset, are derived from β5t-expressing cells (22). Thus, while β5t expression is unique to TEC, and underpins cTEC function, its expression is not exclusive to mature cTEC. Moreover, expression of β5t by TEC progenitors is also relevant to understanding functional heterogeneity in pathways of TEC development. For example, it is not clear whether β5t^+^ progenitors are capable of cTEC functions such as positive selection prior to their transition toward the mTEC lineage. In this scenario, the embryonic thymus may harbor TEC progenitors that go through serial progression development (23), in which β5t^+^ TEC first function as cTEC, and then differentiate further toward mTEC. Alternatively, β5t^+^ progenitors may not possess functional capabilities of cTEC, and represent functionally immature cells that serve as a source of functionally competent cTEC and mTEC. Whatever the case, expression of β5t by both TEC progenitors and mature cTEC makes it difficult to directly define and discriminate between these cell types, particularly in the embryonic thymus.

Although the properties of the thymus cortex are becoming increasingly well-defined, functional heterogeneity within cTEC is still relatively poorly understood. Efforts have been made to investigate the cTEC population, and heterogeneity has been suggested using Sca-1 and α6-integrin, with Sca-1 positive cells expressing high levels of MHCII (20). Some of the difficulties in investigating the cTEC population is likely due at least to the relative low yield of cells obtained from the thymus compared to mTEC, making cTEC a difficult population to study. However, a recent study bypassed this problem by analyzing cTEC in mice in which CyclinD1 is overexpressed by K5 expressing cells. Although TEC were found at ~100-times greater frequency, the thymus from these mice had a normal structure, and supported a normal program of T cell development. Mass spectrometry proteomics and single cell RNA sequencing confirmed cTEC specific expression of Cathepsin L, TSPP, and β5t, and mTEC-specific expression of Cathepsin S, CD40, and Aire (24). These mice could prove to be a useful platform for further interrogation of the cTEC compartment.

In relation to functional heterogeneity within cTEC, thymic nurse cells (TNC) are large epithelial cell complexes in which single cTECs encase viable thymocytes. This is a unique feature of cTEC, and ~10–15% of cTEC form such complexes; each containing between four and eight DP thymocytes (24). TNC are absent from the embryonic thymus, and are only detectable from 5 days post-birth; perhaps a reflection of a bias toward mature cTEC rather than cTEC-like progenitors. In addition, cTECs that form TNC have increased expression of CD205, CXCL12, TGFβ, TSSP, and VCAM-1, compared to cTEC that are not part of TNC structures (25). Interestingly, thymocytes contained within TNC are enriched for those that have undergone secondary TCRα rearrangements, suggesting they may provide an environment then enables efficient positive selection. The presence of such epithelial-thymocyte complexes results in an estimated 20% of RNA isolated from total cTECs reflecting gene expression by enclosed DP thymocytes (24). This can be seen in single cell RNA sequencing of cTECs where newborn and adult cTECs appear to be contaminated with DP thymocytes (26). Recent single cell RNA sequencing analysis has begun to describe some heterogeneity within cTEC beyond TNC. For example, Bornstein et al. (9) found two clusters within cTEC with differential expression of genes including *Dll4*. Whether these cTEC differ in their functional capacity has not yet been tested.

Interestingly, constitutive autophagy is a feature of TECs, which contributes to the processing and presentation of MHCII associated peptides. Comparison of this process in cTEC and mTEC using GFP-LC3 transgenic mice to allow the detection of autophagosomes, showed that cTEC exhibit a higher frequency of autophagy-positive cells compared to mTECs (27). The function of this TEC specific feature is not clear, with conflicting reports in the literature. An initial study used *Atg5*^−/−^ embryonic thymus lobes, grafted into nude mice, and showed that host mice generated symptoms of systemic autoimmunity (27). This was challenged by a later study, using targeted deletion of Atg7 in TECs using a K14^Cre^ mouse line. These mice showed an absence of autoimmunity, even when aged (28). Cross comparisons are difficult between both studies due to the differing methods used to delete gene expression from TECs and analyze autoimmunity, thereby highlighting an area of research in need of further clarification.

### mTEC Stem Cells

**Figure 1:**
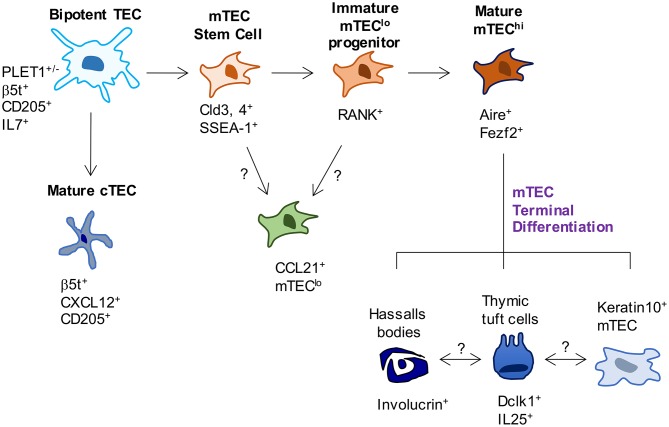
Phenotypic markers and pathways in TEC development. In current models of TEC development, bipotent TEC progenitors with a cTEC-like phenotype give rise to both cTEC and mTEC lineages. Events that occur between bipotent TEC and the generation of mature cTEC are not known. In contrast, SSEA-1^+^ mTEC stem cells have been reported to mark the emergence of the mTEC lineage. While these cells have been shown to give rise to Aire^+^ mTEC, whether they are able to give rise to all currently known mTEC subsets has not been examined. Most relevant to this, the origins of CCL21^+^ mTEC that also reside within mTEC^lo^ are not known, and their status as either immature progenitors or a functionally mature mTEC^lo^ subset requires further study. Downstream of Aire^+^ mTEC^hi^, a terminal differentiation process occurs which gives rise to several TEC subsets and structures, the inter-relationships and functional properties of which remain to be fully determined.

As TEC development involves the formation of distinct cTEC and mTEC populations (Figure 1), several studies have examined early timepoints in the development of these sublineages that are downstream of bipotent progenitors. For example, TEC expression of the tight junction components, Claudin-3 and Claudin-4 (Cld3, 4) has been reported to mark the emergence of the mTEC lineage, with cells expressing these markers giving rise to Aire^+^ mTEC (29). Moreover, a small population of TEC which co-express Cld3, 4 along with the stem cell marker SSEA-1 have been termed mTEC stem cells due to their self-renewal capabilities and their ability to give rise to mTEC but not cTEC. mTEC stem cells have been further characterized by low expression of β5t and CD205, and high expression of RANK and LTβR, and although they are capable of producing downstream mTEC populations in adult thymus, they do so with greatly reduced efficiency compared to in the embryonic thymus (30, 31). Despite expressing very low levels of β5t protein, fate-mapping experiments show these cells have a history of β5t expression, in keeping with them being downstream of β5t^+^ TEC progenitors (31). Interestingly, mice deficient in the master TEC transcription factor Foxn1 have normal frequencies of mTEC stem cells (32), suggesting that the defects in TEC development present in nude mice is downstream of these cells. Importantly, Baik et al. (32) also clarified the function of the TNFRSF member Relb during stages of mTEC development. Although *Relb*^−/−^ mice had unaltered numbers of mTEC stem cells, using RANK^Venus^ reporter mice, it was shown that in the absence of Relb, Cld3, 4^+^ mTEC fail to upregulate RANK expression. The importance of RANK signaling for the mTEC compartment is clear from the large reduction in mTEC, including Aire^+^ mTEC in *Rankl*^−/−^ mice (33). Although the generation of mTEC stem cells does not require Foxn1 or Relb, it has been shown at least in neonatal mice, to be partially dependent on LTβR, as mTEC stem cell frequencies are reduced in neonatal K14^Cre^LTβR^floxed^ mice where targeted deletion of LTβR by TEC has been achieved (34). Despite the clear progress that has been made within this field, complex questions remain, which is due at least in part to a wide variety of markers used to identify TEC populations, as well as differing *in vivo* and *in vitro* methods used to assess their lineage potential. Further work is needed to build a more complete profile of relationships between mature TEC compartments and TEC progenitors, and the developmental requirements of each.

### Immature mTEC Progenitors

In order to gain a better understanding of complexity within TEC populations, recent studies have interrogated the mTEC population using single cell RNA sequencing. One such study sorted total “unselected” mTECs, in addition to mTEC expressing specific Tissue Restricted Antigens (TRAs), namely Tspan8 and GP2 protein. To determine the likely developmental progression (10), clustering, and pseudotime trajectory analysis was performed on the single cell RNA sequencing data obtained from these populations. In agreement with other studies, this study highlighted a distinct population of mTEC phenotypically resembling jTECS (35) through their expression of *Pdpn* and lack of expression of Aire. Importantly, such cells were also defined by expression of the chemokine *Ccl21a*, that plays an important role in the recruitment of positively selected thymocytes into the medulla (Table 1). However, it is important to note that not all *Ccl21* expressing mTEC appear to have high *Pdpn* expression (9). Interestingly, predicative analysis by Dhalla et al. (10) suggested CCL21^+^Pdpn^+^ immature mTEC follow a maturation pathway whereby they upregulate Aire expression, followed by expression of TRAs along with high levels of CD80 and CD86. Consistent with this, the gene signature associated with CCL21^+^ mTEC-I are present within the thymus at E14.5 whereas the genes relating to Aire^+^ mTEC-II are not (9). More recent studies examining the developmental pathway of TEC development have used trajectory analysis of large data sets. Such analysis was performed on clusters of jTEC, mTEC^lo^, and mTEC^hi^, identified from single cell RNA sequencing data and supported the previously described immature phenotype of jTEC, and suggested they were most likely to become mTEC^hi^ before downregulating markers associated with maturation to become mTEC^lo^ (36). While these studies provide important new information on mTEC heterogeneity, it is not fully clear whether CCL21-expressing mTEC, that typically lie within the MHCII^lo^CD80^lo^ (mTEC^lo^) compartment represent directly progenitors of later mTEC stages, including mTEC^hi^. Indeed, although immature mTEC progenitors are known to reside within the bulk mTEC^lo^ compartment, the expression of CCL21 by some of these cells suggests that they are already functionally mature (37), and so could be defined as a mature mTEC subset. Perhaps relevant to this, at least in the embryonic thymus, mTEC progenitors that are able to give rise to Aire^+^ mTEC^hi^ can be defined by their expression of RANK (38, 39) (Table 1). Indeed, in both embryonic and adult thymus, RANK itself is a key functional regulator of the maturation of mTEC progenitors into more mature mTEC^hi^ (33, 38–40). Importantly, while RANK expression has relevance to the study of mTEC progenitors, the nature of embryonic mTEC^lo^ progenitors, and their full developmental potential, remains poorly understood. For example, it is not currently known whether RANK^+^ progenitors also express CCL21, a chemokine that is expressed by at least some mTEC (37) or whether RANK^+^ progenitors can give rise to CCL21^+^ mTEC. Moreover, analysis of RANK^Venus^ reporter mice has shown that patterns of RANK expression in the adult thymus are complex, with multiple subsets of mTEC^lo^ and mTEC^hi^, including CCL21^+^ cells and Aire^+^ cells, demonstrating heterogeneity with regard to RANK expression (41). Thus, while it is clear that RANK is expressed by at least some mTEC progenitors, it is not known whether such progenitors have the potential to generate all mTEC subsets. Moreover, RANK may also be expressed by, and operate on, mTEC at other developmental stages. The recent generation of CCL21^tdTomato^ reporter mice (42) together with availability of RANK^Venus^ mice (41) offers a possible way to generate new dual mTEC reporter mouse strains to examine new precursor-product relationships within the mTEC lineage.

### MHCII^hi^CD80^hi^ mTEC^hi^ and Thymic Tolerance

Clear heterogeneity within mTEC exists, however segregation into subsets based on phenotype, developmental requirements and function can make conclusions and cross comparisons challenging. Routinely, mTECs are broadly subdivided into mTEC^lo^ and mTEC^hi^ according to their levels of MHCII and CD80. Perhaps the most defining feature used to discriminate populations within mTEC^hi^ is expression of the autoimmune regulator (Aire). The transcription regulator Aire is required for efficient promiscuous gene expression of TRAs by mTEC, which is vital for the deletion of self-reactive thymocytes. This contributes to the multi-organ autoimmunity in Aire deficient mice and Aire deficient (autoimmune polyendocrinopathy–candidiasis–ectodermal dystrophy) patients. The function of Aire exceeds TRA expression. For example, Aire regulates the expression of XCL1 in mTEC; a chemokine important for the medullary localization of thymic DC and Treg generation. Mice deficient in XCL1 exhibit symptoms of autoimmunity, suggesting that Aire promotes central tolerance via multiple mechanisms (43).

Not all promiscuous gene expression is dependent on Aire, as TRA expression is evident in *Aire*^−/−^ mice. The transcription factor Fezf2, also expressed by mTEC, has been reported to be required for the expression of some Aire-independent genes (44). Parallel to Aire, Fezf2 expression is observed within the mTEC^hi^ population, and moreover immunofluorescent staining reveals co-expression of both Aire and Fezf2 by the same cell (44, 45). Such mTEC^hi^ that express high levels of Aire, Fezf2, and molecules associated with antigen presentation, were also described in a recent single cell RNA dataset and termed mTEC-II (9). Interestingly, co-expression of Aire and Fezf2 can be seen in human mTEC (44, 46), and its expression by Aire^+^ mTEC may suggest that similar mechanisms underpin central tolerance in mouse and man. Importantly, Fezf2 expression has also been observed within mTEC^lo^ (38), suggesting TRA expression is not restricted to mTEC^hi^ cells.

### mTEC Terminal Differentiation and Post-Aire mTEC: Involucrin^+^ Cells

Aire^+^ mature mTEC were once considered to be at the final stages of their maturation, with Aire expression indicative of subsequent apoptosis (47). However, several lines of evidence now show Aire^+^ mTEC can continue their development beyond phases of Aire expression, to become TEC expressing markers typical of terminally differentiated keratinocytes (48, 49). These cells form distinct structures within the thymus medulla termed Hassall's corpuscles, and can be identified based on their expression of keratin-10 and involucrin. TEC with this phenotype are likely to be downstream of Aire^+^ mTEC based on the ontogenetic appearance of both subsets. Aire^+^ mTEC appear first during ontogeny, and are dependent on RANKL provision by DP thymocytes. Subsequently a population of involucrin^+^ mTEC becomes visible, which require LTα expression by positively selected thymocytes for their development (50). This is supported from direct analysis of TEC developmental pathways and fate mapping experiments, which showed that Aire^+^ cells can proceed to become Aire^−^ cells with lower levels of MHCII (49). Further analysis of post-Aire mTEC shows they lack expression of CCL21 (51), making them distinct from other populations of MHCII^lo^ mTEC.

Although the role of Hassall's corpuscles remains elusive, further characterization of the phenotype and function of these cells has been achieved in the human thymus, where these medullary structures are much more prominent. In addition to keratin-10 and involucrin, Hassall's corpuscles in human thymus express filaggrin (52), and thymic stromal lymphopoietin (TSLP) (53). Whilst filaggrin expression within the murine medulla has been demonstrated (54), whether this specifically marks post-Aire mTEC is unclear. Expression of TSLP by Hassall's corpuscles in the human thymus has been shown to induce expression of markers associated with DC activation, which was needed to generate thymic T-Reg (53). This finding was followed up in mice, where TSLP-ZsG reporter lines were used to describe an enrichment of involucrin gene expression within reporter^+^ mTEC (55), however whether there is a role for TSLP expression by terminally differentiated mTEC in the generation of T-Reg in mouse thymus is unknown.

### mTEC Terminal Differentiation and Post-Aire mTEC: Thymic Tuft Cells

A combination of fate mapping experiments and single cell RNA sequencing analysis from two independent groups suggests there are two main populations of post-Aire mTEC (9, 11). One population is the Keratin-10^+^ involucrin^+^ mTEC discussed above, whereas the other is a distinct population of TEC which resemble tuft cells that have been described at mucosal sites. Tuft cells are a type of chemosensory epithelial cell, most studied for their role in controlling helminth infection via activation of ILC2. Comparison of cells isolated from different tissues showed that tuft cells from the thymus had the greatest number of differentially expressed genes compared to tuft cells from other sites (56). Despite these differences, tuft cells from intestinal and thymic tuft cells share similarities, e.g., expression of IL25, Trmp5, Dclk1, and IL17RB.

The functions of these newly defined TEC are yet to be thoroughly explored. Unlike intestinal tuft cells, thymic tuft cells express high levels of MHCII (11) perhaps indicating an active role in antigen presentation and thymic selection. Interestingly however, evidence from Miller et al. (11) indicated a role for thymic tuft cells in central tolerance, through their expression of the tuft cell specific gene IL25. Thus, transplantation of tuft cell deficient *Pou2f3*^−/−^ thymic lobes into nude mice, resulted in the generation of anti-IL25 antibodies upon immunization, suggesting that tuft cells may act as an important source of antigen within the thymus (11). Perhaps also relevant to possible functional significance of thymic tuft cells it is interesting to note that in the gut, activation of tuft cells can be mediated by the microbial metabolite succinate. However, expression of the succinate receptor Sucnr1 is higher in small intestinal tuft cells compared to thymic tuft cells (56), and it is currently unknown whether thymic tuft cells undergo activation, and if so, how this might occur. Initial reports also suggest thymic tuft cells are capable of regulating innate immune networks within the thymus. Pou2f3 is the master regulator of tuft cell development, and as such, *Pou2f3*^−/−^ mice have been used to begin to determine the role of thymic tuft cells, and both thymic ILC and iNKT cells have been examined in tuft cell deficient mice. Bornstein et al. (9) proposed that due to the restricted thymic expression of IL25 by tuft cells, hematopoietic cells expressing IL25R may be dysregulated. In keeping with this, increased frequencies of ILC2 were present in the thymus of *Pou2f3*^−/−^ mice, however whether this is linked to absence of IL25 is not known. Interestingly, analysis of ILC subsets during thymus ontogeny revealed dynamic changes in their makeup. For example, while ILC3 are dominant in the embryonic thymus, ILC2 dominate post-natally (57). The reasons for this developmental switch in intrathymic ILC frequency is not clear. However, it is interesting to note that like ILC2, tuft cells also emerge post-natally, which together with the localization of both cell types within the medulla, may further emphasize a potentially important link between these cell types. If thymic tuft cells are regulators of ILC2, any increase in the latter in tuft cell deficient *Pou2f3*^−/−^ mice would indicate that tuft cell products may act to limit ILC2 proliferation and or survival. While such factors remain unknown, as is the functional relevance of tuft cell control of ILC2 availability, it is perhaps important to note that ILC2 represent an intrathymic source of IL13 (57), a cytokine ligand for the type 2 IL4R that has been shown to be an important regulator of thymus emigration (58). Whether tuft cells limit ILC2-derived IL13 availability that then influences rates of thymus exit, has not been addressed. In addition to tuft cell-ILC interactions, Miller et al. (11) also examined *Pou2f3*^−/−^ mice to assess the potential impact of tuft cells on iNKT cells that represent a non-conventional αβT-cell lineage that is restricted to the non-polymorphic MHC class I like molecule CD1d. In line with a requirement for mTEC in NKT-cell development (59), *Pou2f3*^−/−^ mice were reported to have decreased frequencies of Tbet^+^ NKT1, PLZF^+^ NKT2, and Rorγt^+^ NKT17 within the thymus (11). Interestingly, a reduced frequency of Treg progenitors was also seen in the thymus of both *Pou2f3*^−/−^ mice and iNKT-cell deficient *Cd1d*^−/−^ mice (60). The cellular and molecular interactions that connect tuft cells and iNKT-cells to the intrathymic development of Treg requires further investigation. Finally, studies demonstrating links between tuft cells and iNKT-cells are important as they indicate the importance of mTEC heterogeneity extends beyond its influence on conventional αβT-cell development in the thymus. How tuft cells control distinct subsets of iNKT-cells is currently not known. Given the patterns of IL25R expression by iNKT-subsets (61), and the selective intrathymic production of IL25 by tuft cells, one possibility that requires further examination is that tuft cells directly regulate at least some iNKT subsets, including NKT1 and NKT17 cells that express IL25R, via their provision of IL25. Whether tuft cells and/or additional mTEC subsets have the ability to influence individual iNKT subsets also requires further investigation.

Significantly, while there is evidence for the existence of DCLK1^+^ tuft cells within the human thymus (9), whether human and mouse thymic tuft cells express a similar array of receptors and secreted factors is currently unknown. This will be important to consider when the functions of thymic tuft cells are more fully understood. While immunofluorescence staining of mouse thymus sections showed that both Keratin 10^+^ mTEC and tuft cells are in close proximity to one another (11), the developmental relationships between the two populations is not clear. Although both populations can transit through an Aire-expressing stage, fate mapping experiments showed this isn't a feature of all tuft cells (11). Moreover, the requirement for Aire in their development may differ, for example, *Aire*^−/−^ mice show significantly reduced frequencies of Keratin-10^+^ mTEC (11, 51), whereas tuft cells are present in equivalent numbers in *Aire*^−/−^ mice (11). The initial description of thymic tuft cells proposed Hipk2, an Aire binding partner, to be a molecular regulator of this population, and the generation of Foxn1^Cre^Hipk2^floxed^ mice revealed reduced frequencies of thymic tuft cells (11), however the mechanism behind this is unknown. An additional regulator of Keratin-10^+^ mTEC development is LTα from positively selected thymocytes, as these terminally differentiated mTEC were found in reduced frequencies in *Lta*^−/−^, *Ltbr*^−/−^, and *Zap70*^−/−^ mice (50). Whether lymphotoxin signaling is also a regulator of tuft cell development in the mTEC lineage is yet to be determined.

## Common Origins of cTEC and mTEC

### Bipotent TEC Progenitors

Despite the differing roles of cTEC and mTEC in the adult thymus, their development begins in the embryonic thymus from a common bipotent precursor that gives rise to both lineages. Initial experiments using purified populations of TEC and antibodies against MTS20 and MTS24 showed that both cTEC and mTEC are generated from Placenta expressed transcript-1^+^ (PLET1^+^) TEC (62, 63). However, at this time it was unclear whether a bipotent progenitor or individual cTEC and mTEC-restricted precursors were contained within this fraction. Direct evidence was subsequently demonstrated for the existence of a bipotent TEC progenitor in the embryonic thymus. This was shown by the microinjection of a single EpCAM1^+^ YFP^+^ cell into a non-YFP embryonic thymus, which was then grafted under the kidney capsule of a wildtype (WT) mouse. These grafts contained both Ly51^+^ cTEC, and Keratin-5^+^ mTEC, each anatomically segregated into distinct compartments, thus demonstrating that one cell can produce both TEC lineages (64). Similar conclusions were drawn from an independent study published at the same time that used mice in which a mutant allele of Foxn1 could be reverted to wildtype function in single cells at post-natal stages. Following spontaneous induction of Foxn1 gene expression in a single cell, mice were able to generate thymic tissue containing both cTEC and mTEC providing evidence that bipotent TEC progenitors are also present within the post-natal thymus (65). Although both of these studies highlighted the existence of a bipotent TEC progenitor, the phenotype of such cells remains elusive, despite attempts of further characterization. Using reaggregate thymic organ cultures (RTOC) with purified populations of TEC, Rossi et al. (66) confirmed bipotent TEC progenitor potential by MTS24^+^ TEC, but in addition showed equivalent capabilities within the MTS24^−^ fraction. These findings support the notion that bipotent TEC progenitors express PLET-1, but shows additional progenitors are also present within the embryonic thymus which lack PLET-1 expression. The bipotent potential of PLET-1 expressing TEC isolated from adult thymus has been also assessed by grafting RTOC into WT mice. These grafts showed that UEA-I^−^Ly51^+^PLET-1^+^ cells with high expression of MHCII can give rise to both cTEC and mTEC, and continue to do so up to 9 months later (67).

Beyond these original descriptions of bipotent TEC progenitors, several studies have searched for evidence that supports the existence of bipotent TEC progenitors in the post-natal and adult thymus. For example, studies using long-term BrdU retention (indicative of a quiescent state) in adult TECs revealed MHCII^lo^α6^+^Sca-1^+^ cells at the corticomedullary junction (CMJ) could generate both cTEC and mTEC lineages in reaggregate grafting experiments (68). However, it is not clear whether bipotent TEC or lineage-specific precursors were contained within this fraction, and their low expression of MHCII makes this population distinct from the PLET-1^+^ cells described previously (67). In the embryo, further characterization of bipotent TEC progenitors has been possible, and studies have collectively described a cTEC-like phenotype of such cells using a variety of methods. For example, embryonic mTEC were shown to arise from TEC expressing markers commonly associated with cTEC, e.g., CD205 (69) and IL7 (70). In addition, Ohigashi et al. (22) fate mapped β5t expression; the proteasome subunit expressed by cTEC but not mTEC, and showed that cells with a history of β5t expression later bore hallmark features of mTEC, including Aire expression. In a subsequent study using inducible β5tCre GFP mice to fate-map cells at post-natal stages, Ohigashi et al. (31) showed <5% of mTEC were labeled when mice were treated with doxycycline after 1 week of age, whereas doxycycline treatment from E0 labeled ~80% of mTEC. These results indicate that post-natal mTEC are derived from cells which express β5t embryonically. The shared expression of several markers e.g., CD205, Ly51, β5t, between cTEC and bipotent TEC progenitors makes the respective populations difficult to distinguish. However recent data from embryonic and adult TEC suggests differences now exist, and RNA sequencing analysis shows that embryonic TEC are enriched for genes involved in cell cycling and have a downregulation of genes involved in antigen presentation (9, 26). Such studies may support future approaches to identify differentially expressed genes that help to define and isolate TEC and TEC progenitor subsets.

Additional studies have provided information regarding the anatomical positioning of TEC progenitors in the post-natal thymus. In agreement with the description of an early cTEC phenotype of TEC progenitors, a combination of inducible fate mapping and confetti mice showed clusters of post-β5t expressing TEC that were concentrated at the CMJ (jTEC), which progressed to become mTEC (71). In addition, single cell RNA sequencing datasets show populations that resemble the immature jTECS described here (9, 10). Combined, this data supports a model of serial progression, whereby bipotent precursors acquire traits usually associated with cTEC, before bearing hallmark features of mTEC. Interestingly, cells within the adult thymus resembling stem cells have been described, with Ucar et al. (72) identifying cells capable of forming spheroid colonies typical of cells with stem cell properties. Such colonies, termed thymospheres, lacked expression of EpCAM1 and Foxn1, and were shown to have the capacity to generate both cTEC and mTEC. A more recent study revisited this issue, including the nature of thymosphere forming cells, and showed using a combination of fate mapping mouse strains that thymosphere forming cells are not of epithelial cell origin. Instead, they show by fate-mapping Wnt1^Cre+^ cells, that thymosphere forming cells are neural crest derived, and such structures can incorporate bystander TECs, thereby producing the results seen in the initial study (73). As such, the possible presence and identity of TEC populations with clonal and self-renewing properties remains unclear, and further studies are required to examine the earliest stages in embryonic and post-natal TEC development that give rise to the continual generation of cTEC and mTEC lineages.

## Factors Affecting Rates of Thymus Function

Both chronic and acute damage to the thymus have detrimental effects on its ability to support T cell development. In particular, changes that take place within thymic epithelial microenvironments result in a reduction in T cell production, and such events can take place in several ways. For example, both age-related thymic involution and therapeutic cytoablative treatments erode TEC microenvironments which then impair rates of thymocyte development. Importantly however, regeneration processes can occur within the thymus, and several efforts have been made to understand this process and to harness it for therapeutic benefit.

### Age Related Thymus Atrophy

Natural thymic involution, that occurs as a result of aging, significantly reduces rates of thymic function across the life course. Analysis of recent thymic emigrants as a measure of thymic function in Rag2GFP mice highlights the constant decline in *de novo* T cell production during the first 5–6 months of life (74). Unfortunately, identification of newly produced T cells in humans is more challenging, and currently relies on the PCR quantitation of circular DNA by-products of TCR gene rearrangements termed T Cell Receptor Excision Circles (TRECs), in conjunction with surface markers including CD31 and CD103. Such analysis shows that similar to mice, thymus function in humans also declines with age (75–78), resulting in a pool of peripheral T cells in the elderly which is dominated by clonally expanded cells (79). This impacts on the ability of an aged immune system to respond to challenge such as infection and vaccination.

Age associated thymic atrophy has been well-studied in mice, and attempts have been made to understand the mechanisms behind this phenomenon. The recruitment of T cell precursors into the thymus occurs via blood vessels at the CMJ, and expression of key molecules, e.g., P-selectin and CCL25 are important in this recruitment process (80). Initial reports suggested that expression of these factors is unaffected in aged mice (81), however a more recent publication showed reduced expression of CCL25 in the thymus from aged mice (82). Despite these discrepancies, recruitment of progenitor cells into the thymus does not appear to be the causative factor behind age related thymus involution, as the ability of an aged thymus to recruit intravenous-injected lymphoid progenitors is equivalent to a young thymus (81). Moreover, intrathymic injection of T cell precursors into young and aged thymi show reduced T cell development within the aged thymus (83). Combined, this data suggests that recruitment of progenitors may not be a simple explanation for the limited thymopoiesis in aged mice, but rather that other environmental factors within the thymus likely influence T cell development and thymus cellularity.

Interestingly, analysis of the stromal compartment by immunofluorescence in aged mice showed a progressive loss of both CD205^+^ cTEC and UEA-1^+^ mTEC, resulting in epithelial “free” areas (81, 84, 85). This loss of TEC in aged mice is perhaps caused by reduced levels of proliferation and increased apoptosis (47, 81). Moreover, aged thymi show increased expression of phosphorylated H2AX and p53 binding protein; markers of DNA damage and cellular senescence (84), which could account for the reduced thymus function seen with increasing age. The mechanisms that control TEC proliferation in aged mice is not fully understood, however it is interesting to note that whilst 95% of embryonic TECs express Foxn1, the frequency of Foxn1 expressing TECs decreases post-natally (86). In keeping with reduced Foxn1 expression in thymi from older mice, cTEC from older mice show reduced expression of Foxn1 target genes, e.g., *Dll4, Kitl, Cxcl12*, all of which are important for early stages of T cell development (82). Genetic alterations to Foxn1 in various mouse lines have been used in attempts to understand how Foxn1 may impact age related thymus involution. For example, overexpression of Foxn1 in young mice results in delayed thymus involution (87), whereas reduced Foxn1 expression in young mice causes premature thymus involution (88). In addition, regeneration of thymus function in aged mice has been possible via the upregulation of Foxn1 expression by TEC (82). Interestingly, such studies show Foxn1 aids TEC maintenance and recovery by inducing proliferation of TEC subsets, including MHCII^lo^ TEC that are known to include TEC progenitors.

Proliferation of TEC is required for normal thymus growth during ontogeny, and this high rate of TEC proliferation during early stages of organogenesis is dependent on the transcription factor Myc. Importantly, Myc expression by TEC is high in the embryo but undergoes subsequent downregulation which correlates with age. This important process limits the extent to which the thymus can grow, and therefore may contribute to age-related thymus involution. Cowan et al. (26) induced transgenic expression of Myc by TEC, which maintained a fetal gene signature in adult TEC, and caused excessive TEC proliferation and thymus hyperplasia in adult mice, suggesting this could be a mechanism by which to reverse age related thymic atrophy.

### Recovery of Thymus Function During Immune Reconstitution

**Figure 2:**
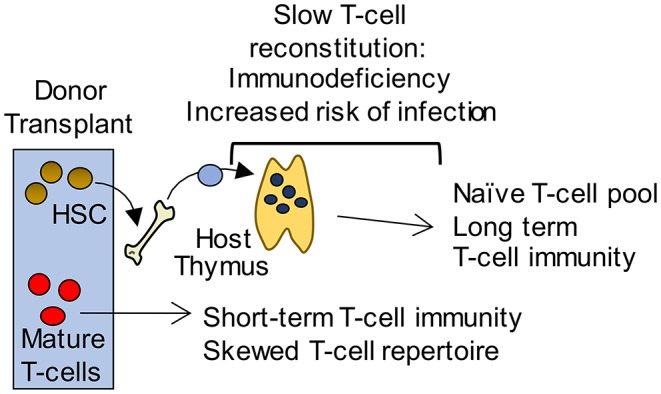
Long-term T-cell immunity following BMT is thymus dependent. Initially, short term T-cell mediated immunity is provided by a donor derived mature T-cell pool with a limited T-cell receptor repertoire that is unable to mount effective immune responses to pathogens. In contrast, a thymus dependent pathway gives rise to an antigenically diverse naïve T-cell pool that provides long term protection. However, this pathway requires graft-derived progenitors to colonize the thymus and undergo T-cell development. As a consequence, T-cell reconstitution is slow and leaves patients immunodeficient and at potentially fatal risk of infection.

Cytoablative therapies such as chemotherapy and radiotherapy, that are often used in conjunction with bone marrow transplant (BMT), cause apoptosis of radiosensitive cells including thymocytes and TEC (89). As a result, there is a diminished capacity for the generation of newly produced naïve T cells. The recovery of T cells in the periphery occurs via two mechanisms; homeostatic expansion of T cells contained within the graft, and the export of naïve T cells from the thymus (Figure 2). The type of conditioning regime along with patient age, is likely to fine-tune the mechanism by which the peripheral T cell pool is restored (90, 91). As in homeostatic conditions, the production of new T cells requires the recruitment of T cell progenitors to the thymus. Thymus reconstitution following BMT appears to be limited by the number of progenitor cells available within the circulation, as a positive correlation is seen between numbers of BM cells administered, and the frequency of donor-derived DP thymocytes (92, 93). For these reasons, most studies have examined the process of thymus homing following BMT.

Thymic epithelial cells, including both cTEC and mTEC are reduced following irradiation in mouse models indicating the radiosensitive nature of these cells (89, 94). Although numbers of TEC are reduced, the ability of such cells to produce chemokines that are important T cell progenitor recruitment, such as CCL19, CCL21 and CCL25 is maintained following irradiation (89). In contrast, endothelial cells, which are important for the recruitment of T cell progenitors, appear to be radioresistant. Interestingly, the presentation of CCL25 by thymic endothelium is transiently disrupted following irradiation which is proposed to limit thymus reconstitution, as pre-treatment of bone marrow cells with CCL25 caused increased T cell progenitor entry to the thymus following irradiation. This is in keeping with the requirement of CCR7 and CCR9 for T cell progenitor recruitment to the thymus during steady state conditions (95), which is mirrored at long-term time points following BMT where CCR7/CCR9 double-deficient bone marrow cells were shown to contribute very minimally to the pool of DP thymocytes. In contrast to this, early thymus reconstitution following BMT does not require CCR7 and CCR9, as T cell progenitors deficient in both chemokine receptors generate DP thymocytes to an equivalent ability to WT cells in a competitive bone marrow chimera model (92). Combined, these results indicate a transient period of time soon after BMT in which thymus settling occurs independently of CCR7 and CCR9. Furthermore, additional regulators of thymus homing in the steady state and following BMT have been identified, including PSGL-1, the ligand for P-Selectin (80, 92), and lymphotoxin beta receptor (LTβR) (96). Within the thymus, LTβR is expressed by TEC, thymic mesenchyme and thymic endothelial cells, and it's expression by endothelial cells is required for thymus homing during homeostatic conditions. This was illustrated by reduced frequencies of early thymic progenitors (ETP) in thymi from germline *Ltbr*^−/−^ and Tek^Cre^LTβR^floxed^ mice (96, 97). Moreover, the importance of LTβR for thymus homing during BMT has been demonstrated by stimulating LTβR using an agonistic antibody during the time of BMT. This resulted in increased thymus and peripheral T cell reconstitution, suggesting that boosting thymus homing via this mechanism can favorably impact the peripheral T cell pool, and could thus pose a potential therapeutic target (96).

The impact of reduced thymus homing following irradiation is long-lived, as mice irradiated using a sublethal dose at 2 months of age, show reduced frequencies of ETP 16 months later (12). This study also showed unaltered expression of CCL25 following irradiation but did not assess CCL25 presentation by endothelial cells. Instead, Xiao et al. (12) propose that long-term effects are seen within LSK that reside within the BM. Quantitation of this population in irradiated mice revealed a decrease in the percentage and number of LSK 7 months post-irradiation, suggesting that reduced numbers of LSK are responsible for reduced ETP rather than reduced recruitment of these cells to the thymus. Importantly, this study showed that despite the reduction in ETP seen following BMT, there was a compensatory proliferation of DN3 thymocytes, and as such thymus cellularity was unaffected. Contradictory to this, Zhang et al. (89) used irradiation which was targeted to the upper or lower half of mice. Such treatment revealed an impact on donor-derived T cell development only when the upper half of the body was exposed to irradiation. Although this study didn't specifically target only the thymus with their method of irradiation, the results would suggest that damage to the thymus is the biggest driver in limiting T cell reconstitution following BMT.

## Approaches to Enhance Thymus Recovery

### IL22 and BMP4

While mechanisms that regulate thymic regeneration following damage remain unclear, several studies have provided some understanding of factors that may regulate this process. Following depletion of DP thymocytes, intrathymic IL-22 levels were found to increase, suggesting a link to mechanisms of endogenous recovery. In support of this, total body irradiation (TBI) of IL-22 deficient mice resulted in defective thymus regeneration compared to WT mice. Furthermore, when irradiation was targeted to the thymus, an increase in IL-22 was also observed suggesting a direct intrathymic recovery mechanism. Interestingly, IL-22 levels were recorded at the highest level when the thymus had the smallest cellularity, suggesting an inverse relationship between levels of IL-22 and thymic size. Production of IL-22 following damage was attributed to radioresistant thymic LTi/ILC3 which were present in increased numbers following thymic insult. Interestingly, irradiated RORγt deficient mice that lack LTi/ILC3 did not increase their levels of intrathymic IL-22 after damage, suggesting a need for RORγt-dependent cells, including LTi/ILC3, during thymus recovery following damage. Importantly, IL-22R is expressed by TEC, and IL-22 increased thymus cellularity due to increased proliferation of TEC and increased frequencies of all developing thymocyte subsets. These positive effects of IL-22 on thymus regeneration is limited to damage, as steady state mice treated with IL-22 showed no increase in total thymus cellularity (98). It is important to note that in this study, following TBI, LTi/ILC3 cells also upregulated RANKL expression, a molecule which has since been implicated in thymus regeneration. In another study (99), and following irradiation of WT mice, CD45^+^ cells upregulated RANKL expression compared to non-irradiated controls. Further analysis showed that RANKL expression was upregulated by radioresistant host LTi/ILC3 and CD4^+^ cells. Although LTi/ILC3 cell numbers in the thymus are low, their expression of RANKL was significantly higher than CD4^+^ SP thymocytes. To confirm the role of RANKL in thymus regeneration, WT mice were subjected to TBI, followed by neutralization of RANKL via antibody blocking that resulted in impaired TEC recovery. In a subsequent experiment, mice subjected to TBI and administered with exogenous RANKL showed significant increase in TECs compared to control mice. Here, exogenous RANKL treatment resulted in increased Ki67 expression by both cTEC and mTEC, indicative of increased proliferation, as well as reduced expression of pro-apoptotic genes. Interestingly, and perhaps in line with the potential importance of additional TNF Receptor superfamily members in thymus regeneration, a role for lymphotoxin signaling was also suggested, as stimulation by RANKL caused induction of LTα expression by LTi/ILC3 cells. Moreover, LTα deficient mice had reduced TEC recovery post-BMT compared to WT hosts, suggesting a mechanism of TEC recovery via LTα upregulation mediated by RANKL (99).

In addition to the potential of IL22, a recent study highlighted the involvement of bone morphogenic protein 4 (Bmp4) in thymus recovery following damage (100). Mice that were subjected to TBI show an upregulation of intrathymic Bmp4 levels suggesting this pathway may also be involved in thymus regeneration. In line with this, inhibition of Bmp4 by a pan BMP inhibitor prior to TBI caused an impairment in the thymus recovery mechanism. Bmp4 is expressed by multiple stromal cell types within the thymus, including fibroblasts and endothelial cells. Analysis of Bmp4 expression by qPCR on sorted populations of stromal cells following TBI revealed that Bmp4 expression was upregulated only by endothelial cells. Ex vivo expansion of EC that were transplanted post-TBI resulted in an increased TEC population, specifically cTEC, and qPCR analysis of cTEC showed an increase in Foxn1 levels as well as the Foxn1 target genes *Dll4, Kitl*, and *Cxcl12*, thus implicating Bmp4 by endothelial cells to initiate thymus recovery. Moreover, tamoxifen induced deletion of Bmp4 in endothelial cells prevented thymus recovery following TBI. Similar to the radioresistance of ILC3/LTi post-TBI, endothelial cells are also proposed to be radioresistant, as their frequencies remained unchanged in the thymus post-TBI.

### Keratinocyte Growth Factor

Thymic GVHD targets the thymic microenvironment and impairs thymopoiesis. However, studies have shown that thymic GVHD can be abrogated by keratinocyte growth factor (KGF) treatment prior to transplant (94, 101–103). In a model of GVHD, mice were transplanted with allogeneic splenocytes and treated with KGF for a period of 3 days prior to and after transplant. Control mice which did not receive KGF treatment showed a reduction in thymus weight and cellularity as a result of thymic GVHD. However, treatment with KGF inhibited the induction of thymic GVHD. Furthermore, overall percentage of donor T cells, specifically CD8^+^ T cells, infiltrating the thymus was shown to be reduced following KGF treatment. Despite this, absolute numbers of cells were not reduced suggesting that abrogation of thymic GVHD by KGF treatment was not due to decreased infiltration of donor transplanted T cells. Analysis of T cell development showed a loss of DP thymocytes in mice with GVHD, which was restored with KGF treatment. Although KGF treatment was able to protect the thymus from GVHD, it did not prevent donor T cell infiltration into the spleen which resulted in acute GVHD. The receptor for KGF (FGFR2IIIb) is expressed by TECs, thus the thymic microenvironment was analyzed post-GVHD induction. Thymic cortex/medulla organization was found to be severely disrupted, however organization was maintained following KGF treatment suggesting KGF acts on TEC to protect thymic microenvironments and subsequently promote thymopoiesis (101).

Other studies have also assessed the impact of KGF treatment in a mouse models that are aimed to mimic clinical settings. Here, mice were pre-conditioned with both irradiation and cyclophosphamide then reconstituted with MHC-mismatched bone marrow. Strikingly, KGF treatment allowed for sustained increased numbers of thymocytes for at least 3 months. In addition, KGF treatment increased frequencies of peripheral donor derived naive T cells, suggesting increased thymic output rather than peripheral T cell expansion (102). Furthermore, when KGF deficient (*Fgf7*^−/−^) mice were sub-lethally irradiated to dissect the role of endogenous KGF on thymus regeneration, they displayed significant reductions in all thymocyte subsets. In addition, *Fgf7*^−/−^ hosts that received allogeneic or syngeneic BM showed impaired regeneration of thymus as well as reduced peripheral donor and host T cells compared to WT hosts, suggesting host KGF is necessary to mediate thymus regeneration post-BMT (103). Similarly, Kelly et al. (94) studied effects of combined treatment of KGF and the p53 inhibitor Pifithrin-β (PFT-β) on thymus recovery. Lethally irradiated mice were reconstituted with T cell depleted BM and treated with KGF, PFT-β, or KGF and PFT-β. Analysis of the TEC compartment 2 weeks post-BMT showed improved thymus recovery in mice receiving combined treatment compared to either KGF or PFT-β treatment alone. Interestingly, TECs co-staining with Ly51 and Keratin5 were seen following combined treatment with KGF and PFT-β suggesting bipotent progenitors may aid in the observed TEC regeneration. Importantly, intrathymic biotin labeling, as a means to measure thymic output, showed that combined treatment was able to improve thymic export (94). In relation to effects of KGF in humans, attempts to restore T cell numbers in relapse-remitting multiple sclerosis (RRMS) patients following antibody mediated-lymphocyte depletion, KGF treatment, given as palifermin, was shown to reduce thymopoiesis as T cell output was measured by naïve T cell count, RTE and TRECs. Strikingly, reduced thymic output was recorded 1 month post-palifermin administration, as numbers of naïve CD4^+^ were reduced compared to the placebo group. In addition, frequencies of RTE were reduced following treatment with palifermin up to 6 months later. CD4^+^ effector memory cells were increased post-palifermin suggesting decrease in TCR repertoire (104). Despite improved thymopoiesis in murine models, KGF treatment in clinical trials has not improved T cell reconstitution, suggesting different requirements for KGF mediated TEC recovery in humans.

## Conclusions

The importance of thymic epithelial microenvironments for T cell development is well-established. Despite this, we still lack a clear understanding of how TEC populations are established during development, and how they change during the life-course. Importantly, we still do not have a clear picture of the changes that take place in TEC populations in response to thymus damage, and how they are restored either naturally or following therapeutic intervention. As such, understanding the cellular makeup of TEC subsets is a key initial step to gain a clearer view of TEC biology, and also inform and improve approaches to manipulate TEC microenvironments with the longer-term goal of boosting immune system recovery. Relevant to this, several studies have now reported previously unrecognized heterogeneity within TEC compartments, and single cell RNA sequencing approaches represent a powerful approach to initially describe new TEC populations. A key goal of future studies will be to examine the potential functional importance of these newly described subsets, and to place them in a developmental sequence that will provide a detailed roadmap of stages in TEC development. For example, the mTEC^lo^ population, that constitutes the majority of mTEC in the adult thymus, is now known to contain multiple populations that include progenitors of mTEC^hi^, CCL21^+^ mTEC, and stages that represent post-mTEC^hi^ cells. Thus, and in contrast to initial thoughts, mTEC^lo^ do not simply represent ‘immature mTEC'. Approaches that enable the isolation and study of individual mTEC^lo^ subsets will be needed to understand the functional importance of this diversity. Relevant to this, gaining a better understanding of the functional properties of recently described thymus tuft cells, that reside within mTEC^lo^ and represent mTEC developmental stages that occur beyond the mTEC^hi^ stage, may be important in revealing how the thymus medulla is able to support the development of multiple T cell lineages that include conventional αβT-cells, Foxp3^+^ T-Reg and CD1d-restricted iNKT cells.

Finally, and as previously noted, while it is clear that TEC recovery occurs in damage settings that include bone marrow transplantation, it is not known whether such recovery involves a complete restoration of all TEC subsets that are now known to exist. Indeed, it is not known whether therapeutic treatments such as IL22, RANKL and KGF impart their effects via TEC progenitors, or via individual or multiple stages in the TEC developmental program. Again, understanding the relationships between newly described TEC populations, and identification of the factors that control their development, survival and expansion will be an important step in optimizing approaches to target TEC populations for therapeutic benefit.

## Author Contributions

AA and BL reviewed the literature. AA, BL, and GA wrote and edited the manuscript.

## Conflict of Interest

The authors declare that the research was conducted in the absence of any commercial or financial relationships that could be construed as a potential conflict of interest.
